# Identification of differentially expressed genes from *Trichoderma harzianum* during growth on cell wall of *Fusarium solani* as a tool for biotechnological application

**DOI:** 10.1186/1471-2164-14-177

**Published:** 2013-03-15

**Authors:** Pabline Marinho Vieira, Alexandre Siqueira Guedes Coelho, Andrei Stecca Steindorff, Saulo José Linhares de Siqueira, Roberto do Nascimento Silva, Cirano José Ulhoa

**Affiliations:** 1 Departamento de Bioquímica e Biologia Molecular, Instituto de Ciências Biológicas, Universidade Federal de Goiás, Campus Samambaia, P.O. Box 131, Goiânia, GO CEP 74001-970, Brazil; 2 Escola de Agronomia, Universidade Federal de Goiás, Campus Samambaia, P.O. Box 131, Goiânia, GO, CEP 74001-970, Brazil; 3 Departamento de Bioquímica e Imunologia, Escola de Medicina, Universidade de São Paulo, Ribeirão Preto, SP, 14049-900, Brazil

**Keywords:** *T. harzianum*, *F. solani*, Subtractive library hybridization, Gene expression, Mycoparasitism

## Abstract

**Background:**

The species of *T. harzianum* are well known for their biocontrol activity against many plant pathogens. However, there is a lack of studies concerning its use as a biological control agent against *F. solani*, a pathogen involved in several crop diseases. In this study, we have used subtractive library hybridization (SSH) and quantitative real-time PCR (RT-qPCR) techniques in order to explore changes in *T. harzianum* genes expression during growth on cell wall of *F. solani* (FSCW) or glucose. RT-qPCR was also used to examine the regulation of 18 genes, potentially involved in biocontrol, during confrontation between *T. harzianum* and *F. solani*.

**Results:**

Data obtained from two subtractive libraries were compared after annotation using the Blast2GO suite. A total of 417 and 78 readable EST sequence were annotated in the FSCW and glucose libraries, respectively. Functional annotation of these genes identified diverse biological processes and molecular functions required during *T. harzianum* growth on FSCW or glucose. We identified various genes of biotechnological value encoding to proteins which function such as transporters, hydrolytic activity, adherence, appressorium development and pathogenesis. Fifteen genes were up-regulated and sixteen were down-regulated at least at one-time point during growth of *T. harzianum* in FSCW. During the confrontation assay most of the genes were up-regulated, mainly after contact, when the interaction has been established.

**Conclusions:**

This study demonstrates that *T. harzianum* expressed different genes when grown on FSCW compared to glucose. It provides insights into the mechanisms of gene expression involved in mycoparasitism of *T. harzianum* against *F. solani*. The identification and evaluation of these genes may contribute to the development of an efficient biological control agent.

## Background

*Trichoderma harzianum* is a soil-borne filamentous fungus that protects crop plants from attack by a range of pathogenic fungi [1]. Species of the genus *Trichoderma* are widely known for their biotechnological interest, however their use as biocontrol agents requires a comprehensive analysis of the biological principles of their action. Their antagonistic abilities are described as a combination of several mechanisms, including nutrient competition and direct mycoparasitism, which involves the production of antifungal metabolites and cell wall-degrading enzymes [2-5]. The use of these species as biocontrol agents represents an environmentally friendly alternative to chemical fungicides, and furthermore, some of their genes have been used to improve plant resistance to pathogens and salt stress [6]. Recently, biologically important proteins from *Trichoderma* have been successfully produced for agricultural and industrial applications [1].

The genus *Fusarium* comprises a wide and heterogeneous group of fungi that causes economically harmful diseases in many crops, such as soybean (*Glycine max*), tobacco (*Nicotiana tabacum*) and common beans (*Phaseolus vulgaris*), reducing both the quality and the quantity of their products [7]. *Fusarium solani* occurs in practically all of the common bean-producing regions in Brazil, and has been controlled through the use of chemical fungicides [8]. Studies on the antagonistic capacity of *T. harzianum* have revealed that it represents an important alternative to the use of chemical fungicides [9]. Comprehensive analysis of the molecular mechanisms used by *T. harzianum* during interaction with *F. solani* is required in order to identify the molecular determinants of its role as a biological control agent [10,11]. The identification of the *Trichoderma* genes involved in these mechanisms and analysis of their expression profiles can provide researchers with biotechnological tools that exhibit anti-fungal activity and that could potentially be used as transgenes capable of inducing resistance to pathogens in economically valuable plants.

The aim of the present study was to provide helpful insights into the mechanism of *T. harzianum* in its action against *F. solani*. We used a suppression subtractive hybridization (SSH) approach to obtain genes that are differentially expressed during *T. harzianum* growth on *F. solani* cell wall or glucose. We analyzed the differentially-expressed genes for homology and classified them into functional categories. Finally, we discuss the possible functional roles of the genes identified in the interaction between *T. harzianum* and *F. solani* by using quantitative real-time RT-PCR.

## Results and discussion

### Identification of differentially expressed genes during growth of *T. harzianum* in FSCW or glucose

In this study, a suppression subtractive hybridization (SSH) approach, which is an efficient method for the isolation of differentially expressed genes, was used to isolate and identify genes that are differentially expressed during *T. harzianum* growth on FSCW or glucose. Samples of mRNA from four incubation times (24, 36 and 48 h) were used to construct a unique cDNA library. In order to obtain a cDNA library enriched for sequences representative of those genes up-regulated in the presence of FSCW-library, cDNA from *T. harzianum* grown in FSCW was used as the tester and cDNA from *T. harzianum* grown in glucose medium as the driver (Glc-library). The Glc-library was enriched with possible genes down-regulated during *T. harzianum* growth on FSCW, a mycoparasitism-related condition. The approach based on the construction of cDNA libraries from a mixture of conditions was previously used successfully in *T. harzianum*[10,12].

In the FSCW-library, 417 reads were generated, which were grouped into 77 Unigene clusters (representing 39 contigs and 38 singletons), with an overall EST redundancy of 91%. The inspection of these Unigene clusters detected matches for 64% using blastx. Seventy eight reads were generated from the Glc-library, which represented 47 Unigene clusters, including 19 contigs and 28 singletons, with an overall EST redundancy of 64%. Among these clusters, 57% presented sequence similarity with GenBank entries using the blastx algorithm. The comparison between the sequences from both libraries indicated that there was no overlap between them, suggesting that the subtraction approach was successful and that the two libraries were in fact enriched for sequences from genes differentially expressed under each condition. All ESTs were submitted to GenBank (accession numbers from JK840901to JK841024).

### Functional annotation of the differentially expressed genes

**Figure 1:**
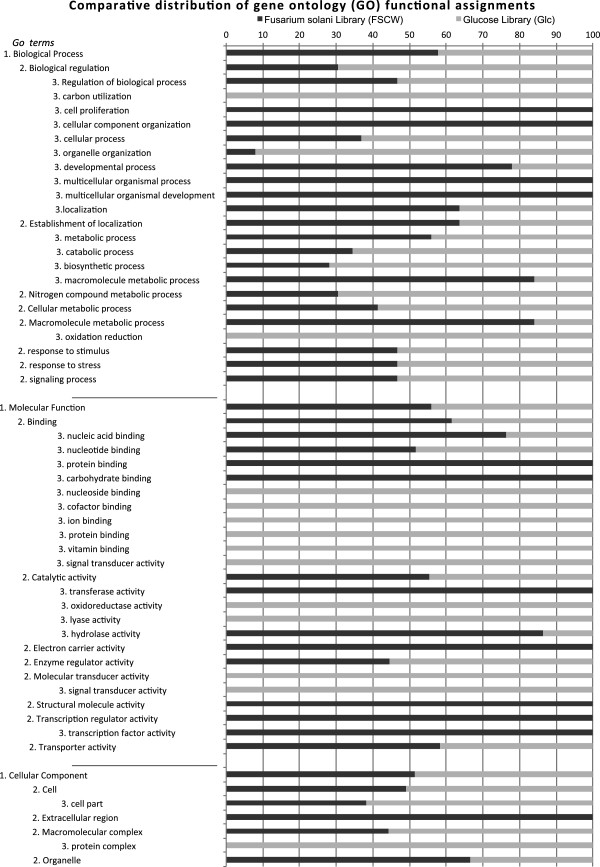
Comparative distribution of ESTs from FSCW and glucose libraries within the gene ontology (GO) functional assignments.

ESTs were annotated according to Gene Ontology (GO) guidelines (Ashburner et al. 2000) with Blast2GO, a universal web-based annotation application [13]. Genes from both libraries were allocated to the main GO categories and the distribution of ESTs in these categories was taken as a measure of gene concurrence across the two libraries (Figure 1). For this purpose, the total number of unique sequences from the two libraries that possessed an assigned GO term within each of the three organizing principles of GO (Biological Process, Molecular Function and Cellular Component) was taken as 100%. Functional annotation of the genes from the FSCW-library indicated that the highest percentage of GO terms was seen in the categories from Biological Processes: localization (40%), cellular process (40%), and metabolic process (64%), as well as the categories related to Molecular Functions: binding (51%), transporter activity (26%), catalytic activity (62%), and hydrolase activity (29%). The GO terms related to regulation: biological regulation (4%), enzyme regulator activity (4%), and transcription regulator activity (4%) as well as proliferation: cell proliferation (4%), demonstrated the lowest percentage values. Genes involved in sphingolipid metabolism, N-glycan biosynthesis, glycosaminoglycan degradation, glycosphingolipid biosynthesis, other glycan degradation, oxidative phosphorylation, amino sugar and nucleotide sugar metabolism, amino acid and water transport, hydrolytic activity and energy related processes were identified during *T. harzianum* growth in FSCW. Moreover, proteins found to be associated with the response of *T. harzianum* to the presence of phytopathogens included: MAP kinases (serine-threonine protein kinase and *chk*1), enzymes that are essential for maintenance of the cell wall integrity, hyperosmotic stress tolerance proteins, pathogenicity factors, a serine protease and a QID 74 protein considered to be involved in the mycoparasitism [14-16]. Annotation and KEGG analysis of the identified sequences demonstrated a clear relationship with the biological processes and molecular functions required during mechanism of biocontrol [1,5].

Functional annotation of the sequences from the Glc-library identified genes belonging to the following categories of Biological Processes: cellular process (94%), cellular metabolic process (63%), metabolic process (69%), catabolic process (31%), biosynthetic process (56%), and also to the following categories from Molecular Functions: catalytic activity (65%) and lyase activity (35%). KEGG analysis showed that the major metabolism pathways corresponded to biosynthetic pathways: glycerophospholipid metabolism, and glycolysis/gluconeogenesis. This fact is consistent with the extensive metabolic activity expected for a filamentous fungus growing on a rich medium with an easily assimilable substrate [17].

### Expression analysis of genes from *T. harzianum* during growth in FSCW

Amongst the differentially expressed genes identified in the SSH analysis, twenty-eight genes were selected based on their predicted function or involvement in *Trichoderma* development, metabolism and biocontrol activity (Table 1). All of these genes were further analyzed by quantitative real-time RT-PCR (RT-qPCR) in order either to validate the results obtained by the SSH method or to understand the kinetics of their expression in the evaluated conditions. We then extended the studies on the expression of these genes by evaluating samples obtained at 24, 36 and 48 hours during growth of *T. harzianum* on FSCW (Table 2 and Additional file 1). The RT-qPCR results suggest that the genes identified as altered in the FSCW-library and annotated as hydrolases (acid sphingomielinase (*asm)*, β-1,3-endoglucanase (*bgn)*, chitinase 33 (*chit*), endochitinase 42 (*endo*), exo-rhamnogalacturonase (*exo*) and glycosyl hydrolase (*glyc*)) were up-regulated with the highest expression values at 24 hours with a statistically significant decrease on these values at 36 and 48 hours of growth. Two other genes identified in the FSCW-library which were annotated in the catalytic activity category (amine oxidase (*aoc*) and phospholipase d (*pld*)), were down-regulated in all times of growth (p < 0.01). Genes associated with binding activity such as checkpoint-like protein (c*hk1*), serine threonine-protein kinase (*sck1*) and senescence-associated protein (*sag*) presented the highest expression values at 24 hours and a decrease on these values at 36 and 48 hours of growth (Table 2 and Additional file 1). The observed expression of mannose-binding lectin (*mbl2*) increases over time (Table 2 and Additional file 1).

**Table 1:** List of genes selected for differential expression analysis and the oligonucleotides used in this study

**Putative function**	**Accessionnumber**	**E-Value**	Protein ID*	**qPCR forward (F) and reverse (R) primers (5' to 3')**
acid sphingomielinase (*asm*)	JK840922	1.39E-136	548323	Forward: GCGAAGCATCTCGGCTATTGTAGT
				Reverse: TCAAGTTGTGAACCGCTACTCGTC
β-1,3-endoglucanase (*bgn*)	JK840920	8.92E-21	241696	Forward: TCAACATCGCCAACGTCAACGAC
				Reverse: TGCCAATACGGGAACCAGTGATC
chitinase 33 (*chit*)	JK840912	3e-51	387920	Forward: TGGAGCTCAACAGGCGCTGC
				Reverse: ACGACGGCACTGCCAAAGGG
endochitinase 42 (*endo*)	JK840909	3.0E-46	364419	Forward: AAGGGTTACTACAGCTACAACGCC
				Reverse: ACTTGAGGTAGGCAACCTTGGTGT
exo-rhamnogalacturonase (exo)	JK840947	2.12E-65	463001	Forward: TTACCTGAAGACATGGGCGGGAAT
				Reverse: GCCTTCCGCCAATCAGCTTAACAT
glycosyl hydrolase (*glyc*)	JK840945	1.51E-09	199282	Forward: GAAATGTTGTCGTCACCAGACGGT
				Reverse: GGCCGCGATTGCTGTTTCATAGT
amine oxidase (*aoc*)	JK840953	9.50E-15	538657	Forward: ATACACCCGAAGGAACCTTGTTGG
				Reverse: TAGCGTGCCTCAATCTCCTTAGCA
phospholipase d (*pld*)	JK840907	3.58E-56	537712	Forward: TGGGAAGACGTTGCACACACAAAC
				Reverse: AAATTGTCGTAGTCGTCCCAGGTG
checkpoint-like protein (*chk1*)	JK840936	2.48E-14	NA	Forward: TGCTGCCTTCCTTGGATGTAGTAG
				Reverse: AAACATGGTGGCAACGGGTAACG
serine threonine-protein kinase (*sck1*)	JK840919	1.15E-80	480202	Forward: ATGCTGAAGAGCTTAAACGCCACC
				Reverse: ACTTTGGCTTGAAGGGTGGAGAG
senescence-associated protein (*sag*)	JK840934	5.02E-30	547464	Forward: AGCTCACGTTCCCTATTAGTGGGT
				Reverse: ATCCTTCGATGTCGGCTCTTCCTA
mbl2-like secreted (*mbl2*)	JK840948	2.05E-41	257052	Forward: TTGCTACGAGGGAGTTTGTTCCTG
				Reverse: TGGAGTTGCACTGGTCTGAAGT
serine protease (*ser*)	JK840930	1.54E-12	366985	Forward: TGGAAGGGAGTGACCAAGCCTG
				Reverse: GGAAAGGTCAGGAGTGCTATCGGG
aquaporin (*aqp*)	JK840978	1.47E-60	476226	Forward: GTTGATGGCATAACCAGTCTCCCA
				Reverse: CAACAACATTGGAGCCGGAAACCT
duf895 domain membrane protein (*duf*)	JK8409133	1.67E-92	396055	Forward: TCCAATCCTTGCCGACGTAGTTGA
				Reverse: TGCCAAGATCACATGGGTCGTTCT
peptide transporter (*ptr2*)	JK840963	8.11e-74	533699	Forward: AGTCATCTGGTTGTAGGCCAGGAA
				Reverse: AAATTGTCGTAGTCGTCCCAGGTG
QID74 protein (*qid*)	JK840906	6.31E-113	456637	Forward: CAGAAGAAGTGCGTGTGCAACAAG
				Reverse: AGCTAGCATCTTTGCCGCAGTTTG
eight cysteine-containing domain (*cfem*)	JK840940	1.09E-17	245062	Forward: GCGTCCGCAAAGAAACAACCTTCT
				Reverse: AGAGAGCGGTGTTTGTAGCGATGA
Enolase (*eno*)	JK841017	3.54E-12	315824	Forward: ACTTTGACCGAGTCTATCCAGGCT
				Reverse: ATACCGACGGAGATGTCAGCAATG
glyceraldehyde 3- phosphate dehydrogenase (*gapd*)	JK840981	1.32E-32	281265	Forward: CAGGTCGCCAAGAAGGTCATCATT
				Reverse: AAGCGTTGGAGATGACATTGGCAC
pyruvate decarboxylase (*pdc*)	JK840980	2.60E-31	277935	Forward: GCAGGTGTTGGTCAATTCCTTCAG
				Reverse: AACGCCAGATGGGAACTTGGTATC
heat shock protein (*hsp98*)	JK841007	9.91E-13	225475	Forward: TTGAGCGTCGTTTCCAACAGGTTC
				Reverse: TGTCGAGAATGCTGACCTTGTGGT
zinc finger domain protein (*c2h2*)	JK841004	1.67E-15	543717	Forward: CAGACCTTGCACTTGTGCTTCTT
				Reverse: AATGTTGTCGACCTCACTGCCT
hexose transporter-like protein (*ht*)	JK840987	1.20E-35	360713	Forward: GGAGTCCCATTTGCTCGAAGTGAT
				Reverse: CGTGCTCATCGTCTTCTT CTTCGT
zinc-regulated transporter (*zt*)	JK840990	3.84E-15	295941	Forward: GGCCAAGAAATCCAGCAGGAAGAT
				Reverse: TTGACTAGTGGCTGGGCTGAATAC
norsolorinic acid reductase (*norA*)	JK841015	6.41E-37	373869	Forward: ACCGTCTCGTCAACATGAGCTACT
				Reverse: AAGTTCATGGAACCCAAGCACAGC
phosphatidylserine decarboxylase family protein (*psd*)	JK840982	4.91E-23	363281	Forward: TCTTTGAAGGCGTAGGTGATCCGA
				Reverse: GCAGATCCGTAGAGGCAAGTATGT
coproporphyrinogen oxidase (cpox)	JK841003	4.88E-24	364552	Forward: TGATCCAGGCGTGTTCAGTCCAAA
				Reverse: GCAGATCCGTAGAGGCAAGTATGT
α-tubulin	HS574101			Forward: TATCTGCTACCAGGCTCCCGAGAA
				Reverse: TGGTGTTGGACAGCATGCAGACAG

* BLASTX at http://genome.jgi.doe.gov/ using *Trichoderma harzianum* CBS 226.95 as a reference model.

**Table 2:** **Expression values of genes identified in *Trichoderma harzianum *(FSCW and Glc libraries) during growth on cell wall of *Fusarium solani***

**Putative function**	**Times of induction (Mean ± SE)**		
	**24 hours**	**36 hours**	**48 hours**
**Genes identified in the FSCW library**			
*acid sphingomielinase (asm)*	129 ± 24	12.52 ± 4.67	0.67 ± 0.46
*β-1,3-endoglucanase (bgn)*	1578 ± 201	958 ± 15.2	140 ± 65
*chitinase 33 (chit)*	1039 ± 152	567 ± 414	258 ± 212
*endochitinase 42 (endo)*	1134 ± 903	222 ± 173	259 ± 210
*exo-rhamnogalacturonase (exo)*	20 ± 8.07	1.06 ± 0.17	0.66 ± 0.57
*glycosyl hydrolase (glyc)*	273 ± 38.8	3.64 ± 0.40	2.30 ± 1.75
*amine oxidase (aoc)*	0.31 ± 0.077	0.58 ± 0.33	0.13 ± 0.12
*phospholipase d (pld)*	0.23 ± 0.15	0.29 ± 0.17	0.28 ± 0.18
*checkpoint-like protein (chk1)*	7.9 ± 2.49	0.73 ± 0.61	1.73 ± 0.06
*serine threonine-protein kinase (sck1)*	1.47 ± 0.39	0.59 ± 0.13	0.36 ± 0.35
*senescence-associated protein (sag)*	44 ± 13.1	6.13 ± 2.95	1.93 ± 1.23
*mbl2-like secreted (mbl2)*	15.78 ± 7.08	59 ± 49	81 ± 72
*serine protease (ser)*	180 ± 116	269 ± 247	117 ± 76
*peptide transporter (ptr2)*	140 ± 111	229 ± 195	57 ± 37
*aquaporin (aqp)*	102 ± 37	185 ± 27	58 ± 16
*duf895 domain membrane protein (duf)*	7.86 ± 1.5	80 ± 40	55 ± 28
*QID74 protein (qid)*	8.36 ± 2.31	76 ±48	12 ± 5.2
eight cysteine-containing domain *(cfem)*	182 ± 13.4	118 ± 10	11 ± 0.9
**Genes identified in Glc library**			
*Enolase (eno)*	0.01 ± 0.001	0.06 ± 0.02	0.065 ± 0.0008
*glyceraldehyde 3- phosphate dehydrogenase (gapd)*	0.0009 ± 0.0008	0.005 ± 0.003	0.001 ± 0.0005
*pyruvate decarboxylase (pdc)*	0.01 ± 0.008	0.047 ± 0.004	0.007 ± 0.007
*heat shock protein (hsp98)*	0.05 ± 0.022	0.83 ± 0.44	0.13 ± 0.03
*zinc finger domain protein (c2h2)*	0.17 ± 0.0001	0.42 ± 0.05	0.52 ± 0.26
*hexose transporter-like protein (ht)*	0.002 ± 0.001	0.005 ± 0.0006	0.0009 ± 0.0001
*zinc-regulated transporter (zt)*	0.0002 ± 0.0002	0.01 ± 0.01	0.008 ± 0.007
*norsolorinic acid reductase (norA)*	0.07 ± 0.01	0.03 ± 0.02	0.03 ± 0.02
*phosphatidylserine decarboxylase family protein (psd)*	0.04 ± 0.01	0.15 ± 0.05	0.21 ± 0.2
*coproporphyrinogen oxidase (cpox)*	0.12 ± 0.10	0.12 ± 0.11	0.01 ± 0.002

All results were compared to the respective control group (*T. harzianum* grown in glucose media) at the respective time. (±) Represent standard deviation.

The data were analyzed by the 2^-ΔΔCt ^method.

A serine protease (*ser*) transcript was also identified in the FSCW-library and RT-qPCR analysis indicated that, differently from hydrolases, the highest expression value was detected after 36 hours of growth (Table 2 and Additional file 1). Moreover, similar to serine protease expression, three genes annotated in the transport GO category (peptide transporter (*ptr2*), aquaporin (*aqp*) and a DUF895 domain membrane protein (*duf*)), showed the highest expression values after 36 hours of growth (Table 2 and Additional file 1). Finally, RT-qPCR expression analyses were performed with *qid74* and *cfem* (eight cysteine-containing domain), genes that correspond to receptor activity on GO. Their highest expression values were detected at 36 hours and 24 hours of growth on FSCW, respectively.

RT-qPCR expression analyses with 10 genes identified on the Glc-library were also conducted to observe their expression profile during the growth of *T. harzianum* in FSCW. Our data showed that all genes analyzed were down-regulated (p < 0.01) (Table 2 and Additional file 1). Enolase (*eno)*, glyceraldehyde 3-phosphate dehydrogenase (*gapd*) and pyruvate decarboxylase (*pdc*), enzymes that are involved in glycolysis and/or glyconeogenesis pathways, showed similar expression profiles at 24 and 36 hours of growth (Table 2 and Additional file 1). Also, the genes with binding activity, *hsp98* and a *c2h2* (zinc finger domain protein), showed the lowest expression values after 24 hours of growth in FSCW. Moreover, the results obtained with genes corresponding to transporter activity on GO (hexose transporter-like protein (*ht*) and zinc-regulated transporter (*zt*)) demonstrated the same time-course expression profiles as the genes that present binding activity (Table 2 and Additional file 1). RT-qPCR expression analysis of norsolorinic acid reductase (*nor*A), phosphatidylserine decarboxylase (*psd*) and coproporphyrinogen oxidase (*cpox*) demonstrated that these genes are down-regulated during growth of *T. harzianum* in FSCW (Table 2 and Additional file 1). These enzymes are essential in some filamentous fungi in the biosynthetic pathways of aflatoxins, glycerophospholipid metabolism and heme groups [18,19].

### Expression analysis of genes from *T. harzianum* during interaction with *F. solani*

In order to identify genes potentially involved in biocontrol, RT-qPCR was performed using total RNA from dual cultures of *T. harzianum* and *F. solani* in three different interaction stages: before contact, during contact and after contact (Table 3 and Additional file 2). Only the genes identified in the FSCW library were analyzed by RT-qPCR. As a control, a confrontation assay was conducted where *T. harzianum* was challenged with itself. RT-qPCR analysis showed that the genes studied were not expressed when *T. harzianum* was challenged with itself. We have previously reported that direct confrontation assays are a powerful tool to study the phenomenon of mycoparasitism by *T. asperellum and T. harzianum*[10,20].

**Table 3:** **Expression values of genes identified in *Trichoderma harzianum *at different stages of confrontation to *Fusarium solani***

**Putative function**	**Interaction stages (Mean ± SE)**		
	**Before contact**	**Contact**	**After contact**
*acid sphingomielinase (asm)*	3.22 ± 0.13	2.36 ± 0.14	1.02 ± 0.04
*β-1,3-endoglucanase (bgn)*	0.18 ± 0.01	0.60 ± 0.03	163 ± 11
*chitinase 33 (chit)*	0.28 ± 0.01	0.97 ± 0.03	1105 ± 101
*endochitinase 42 (endo)*	4.01 ± 0.12	1.78 ± 0.10	226 ± 26
*exo-rhamnogalacturonase (exo)*	0.60 ± 0.06	1.14 ± 0.12	7.39 ± 1.19
*glycosyl hydrolase (glyc)*	0.88 ± 0.09	0.95 ± 0.00	3.18 ± 0.30
*amine oxidase (aoc)*	0.87 ± 0.09	0.88 ± 0.05	1.46 ± 0.13
*phospholipase d (pld)*	0.99 ± 0.16	0.95 ± 0.07	1.53 ± 0.14
*checkpoint-like protein (chk1)*	1.01 ± 0.03	1.75 ± 0.13	6.01 ± 0.70
*serine threonine-protein kinase (sck1)*	0.70 ± 0.10	1.01 ± 0.09	3.17 ± 0.26
*senescence-associated protein (sag)*	0.88 ± 0.08	1.49 ± 0.08	6.89 ± 0.71
*mbl2-like secreted (mbl2)*	7.88 ± 0.24	2.52 ± 0.19	5.25 ± 0.49
*serine protease (ser)*	1.41 ± 0.06	2.64 ± 0.09	0.20 ± 0.03
*aquaporin (aqp)*	0.33 ± 0.04	0.64 ± 0.14	1.90 ± 0.19
*duf895 domain membrane protein (duf)*	2.97 ± 0.38	3.17 ± 0.18	3.19 ± 0.29
*peptide transporter (ptr2)*	0.44 ± 0.07	0.89 ± 0.14	11.57 ± 1.17
*QID74 protein (qid)*	5.97 ± 0.28	0.85 ± 0.03	1567 ± 157
eight cysteine-containing domain *(cfem)*	0.86 ± 0.04	0.74 ± 0.05	12.22 ± 1.68

All results were compared to the respective control group (*T. harzianum* challenged with itself) at the respective stage. (±) Represent standard deviation.

The data were analyzed by the 2^-ΔΔCt ^method.

During the confrontation assay most of the genes studied were down-regulated before contact, and the expression of these genes occurs only after contact with the host. However, three genes (*asm*, *mbl2* and *aqp*) presented high expression values before contact. Previously works showed that *Trichoderma* species are able to sense the presence of its host and specific genes are expressed already before contact [21]. Interestingly, with the exception of serine protease (*ser*) all genes are up-regulated after-contact, when the interaction has been established (Table 3 and Additional file 2).

We analyzed the expression of three genes encoding cell wall degrading enzymes (CWDE): β-1,3-endoglucanase (*bgn*), chitinase 33 (*chit*) and endochitinase 42 (*endo*). In agreement with previous studies, transcripts encoding these enzymes were highly expressed mainly after contact indicating intense cell wall degradation in this stage [6,10]. The expression of the genes encoding these enzymes was induced by FSCW (Table 2 and Additional file 2). These enzymes could be also induced by metabolites secreted by the host, and is strongly repressed by glucose [22,23]. These genes play a major role in the mycoparasitic activity against the pathogens especially *R. solani, F. oxysporum* and *S. sclerotiorum*[5].

Exo-rhamnogalacturonase (*exo*) and glycosyl hydrolase (*glyc*) annotated as hydrolases were also up-regulated after contact (Table 3 and Additional file 2). Exo-rhamnogalacturonases belong to the group of pectin-degrading enzymes (PDE) and could be involved in cell degradation of *F. solani*. For instance, an endopolygalacturonase (PDE) produced by *T. harzianum* are known to be involved in the cell wall degradation of *Rhizoctonia solani* and *Pythium*[24]. Glycosyl hydrolases are a widespread group of enzymes that hydrolyse the glycosidic bond between two or more carbohydrates, or between a carbohydrate and a non-carbohydrate moiety. A classification system for glycosyl hydrolases has led to the definition of 85 different families, including amylases, cellulases, β-glucanases and chitinases [25]. Thus, the characterization of the glycosyl hydrolase identified in this work needs to be made to show its role in mycoparasitism.

The expression of genes related with amino acids metabolism (*ser, aoc* and *ptr2*) was also analyzed. Serine protease (*ser*) was up-regulated before and during contact, but down-regulated after contact (Table 3 and Additional file 2). These data show that these enzymes play an important role in the early stages of the interaction between *T. harzianum* and *F. solani*. On the other hand, the expression of amino oxidases (*aoc*) and a peptide transporter (*ptr2*) are up-regulated after-contact when the expression of serine protease decreased. Some proteases from *Trichoderma* species have been identified as having biocontrol functions, including aspartyl protease, serine protease and subtilisin-like protease [5,26]. These proteases can take part in the host cell wall breakdown process or act as proteolytic inactivators of pathogen enzymes. Amine oxidases (AO) are a large group of enzymes catalyzing oxidative deamination of amines to form the corresponding aldehydes, hydrogen peroxide and ammonia. AO in filamentous fungi are involved in amino acid metabolism and are described in many fungi such as *Aspergillus oryzae*, *Penicillinium chrysogenum* and *Fusarium oxysporum*[27]. Peptide transport is a universally observed physiological phenomenon in both prokaryotes and eukaryotes cells [28]. This process is characterized by the ability of cells to transport peptides across membranes in an energy-dependent manner. Internalized peptides are rapidly hydrolyzed by peptidases and the resulting amino acids are used for protein synthesis or as alternative sources of nitrogen and carbon. Vizcaíno et al., reported the cloning and characterization of *ThPTR2*, di/tri-peptide transporter gene from *T. harzianum* related to the mycoparasitic process. This hypothesis was supported by the fact that expression of *ThPTR2* was triggered when *Trichoderma* directly interacted with *B. cinerea*[12].

The transcript *asm*, encoding an acid sphingomielinase (ASM), showed the lowest expression value after contact to the phytopathogen (Table 3 and Additional file 2). ASM are a group of hydrolases that cleave sphingolipids, a common component of plasma membranes, and their products can regulate a variety of cellular functions such as proliferation and differentiation [29]. This enzyme could be involved in providing nutrition for *T. harzianum* from *F. solani* cell components.

The transcripts *duf* (DUF895 domain membrane protein), *mbl2* (mbl2-like protein) and *cfem* (eight cysteine-containing domain) identified in the cDNA library were used in order to learn whether proteins located in membrane or outer surfaces of the cell walls are expressed during the interaction between *T. harzianum* and *F. solani*. These proteins are described as involved in recognition, attachment, adhesion and appressorium development key events mycoparasitism [30]. The transcript *duf* and *cfem* are down-regulated before contact and increased their expression values after-contact (Table 3 and Additional file 2). However, the transcript *mbl2*, encoding for a mannose biding lectin, is expressed in the three stages of interaction. This may be due the fact that these proteins are important in coiling and appressorium formation a key step in the mechanism of mycoparasitism.

The transcript *qid74*, encoding the QID74 cell wall protein, is also up-regulated and was strongly expressed after contact. Studies of QID74 in *T. harzianum* CETC 2413 showed that this protein is involved in resistance of hyphae to lytic enzymes and the ability to adhere to hydrophobic surfaces [31]. High expression values of genes of CWDE (*bgn*, *chit* and *endo*) were seen after contact, suggesting that QID74 was produced with the purpose of protecting the cell wall of *T. harzianum*. These proteins could be also be involved in recognition, attachment and formation of specialized structures such as appressoria.

Among all studied genes only one encoding for aquaporin (*aqp*) was highly expressed in the three stages of interaction to *F. solani* (Table 3 and Additional file 2). These proteins mediate rapid and selective flux of water across biological membranes and hence play important roles in the osmoregulation of cells and organisms. These proteins also facilitate transmembrane transporte of small uncharged molecules like polyols, urea, arsenite and many more, thereby playing roles in nutrient uptake [32].

Interestingly, we found a significant expression of the gene *sag* encoding for senescence-associated protein after contact between *T. harzianum* and *F. solani* (Table 3 and Additional file 2). Senescence is the progressive loss of growth potential of mycelium culminating in total cessation of growth when the culture is considered as dead [33]. Some naturally occurring strains of fungi cease growing through successive subculturing [33]. However, this biological mechanism has to be studied in detail to offer an interpretation about the senescence in *Trichoderma* species and its role in mycoparasitism.

Finally, three genes encoding for phospholipase d (*pld*), serine threonine-protein kinase (*sck1*) and checkpoint-like protein (c*hk1*) involved in transduction cascades, protein modification and fungal morphogenesis were studied [34]. The transcript c*hk1* showed the greatest level of expression followed by *sck1* and *pld*, mainly after contact (Table 3 and Additional file 2). The role of these proteins in mycoparasitism must be studied in more detail, because it involves the participation of a wide range of other genes not described in this work.

## Conclusion

Our results provided a step toward the understanding of the mycoparasitic process of *T. harzianum* during its interaction with *F. solani*. However, future studies, aimed at the functional characterization of genes reported here, will help to better define pathways involved in *T. harzianum* interaction with *F. solani*. A better understanding of the expression profiles of these genes could improve *T. harzianum* performance, either by predicting the regulation of the genes involved in the mycoparasitism or by improving their use in biotechnology processes such as transgenic expression in plants.

## Methods

### Fungal strains and culture conditions

*T. harzianum* ALL42 (Enzymology group collection, UFG-ICB) and *F. solani* (EMBRAPA-CNPAF collection) were used in this study. Both fungi were grown on MYG medium containing 0.5% malt extract, 0.25% yeast extract, 1% glucose and 2% agar. Spores from *T. harzianum* were collected in sterile water, centrifuged at 2,000 *g*, washed twice and used as inoculum (10^7^ spores mL^-1^) in minimal medium, containing KH_2_PO_4_ (2 gL^-1^), (NH_4_)_2_SO_4_ (1.4 gL^-1^), MgSO_4_.7 H_2_O (0.3 gL^-1^), CaCl_2_ .2H_2_O (0.3 gL^-1^), supplemented with 0.5% *F. solani* inactivated cell wall (*F. solani* autoclaved at 120°C for 20 min, washed with distilled water and lyophilized) or glucose. The cultures were grown in conical flasks with constant shaking (180 rpm) at 28°C for 12, 24 and 48 h. Mycelia were harvested, washed twice with sterile water, frozen in liquid nitrogen and stored at -80°C until RNA isolation.

### Isolation of RNA, cDNA synthesis and construction of the suppression subtractive hybridization (SSH) libraries

Total RNA from *T. harzianum* mycelia was extracted using the TRIZOL reagent (Invitrogen), and the resulting quality and concentration were checked by formaldehyde/agarose gel electrophoresis and a spectrophotometer. To obtain the “driver” and “tester” cDNA populations, 0.6 μg of mRNA purified at 24 h, 36 h and 48 h periods were pooled for each set of conditions (FSCW and glucose). The PCR-Select cDNA Subtraction Kit (BD Biosciences Clontech, Mountain View, CA) was used to generate two subtracted cDNA libraries enriched for genes up- and down-regulated in *T. harzianum* grown in FSCW, referred to hereafter as the forward and reverse subtracted libraries. The first subtracted library (forward) was produced by the subtraction of the cDNA population from *T. harzianum* grown in FSCW, used as “tester”, from the cDNA population from *T. harzianum* grown in glucose, used as “driver”. The second library (reverse) was obtained by the subtraction of the cDNA population from *T. harzianum* grown in glucose, used as “tester”, from cDNAs obtained from *T. harzianum* grown in FSCW, used as “driver”. The final PCR products obtained corresponded to genes differently expressed during *T. harzianum* grown in FSCW. PCR products were cloned using the pGEM-T Easy vector system (Promega). Positive colonies were picked out and grown in microtiter plates. Plasmids DNAs were prepared from clones using standard protocols.

### DNA sequencing, processing and EST database construction

*T. harzianum* expressed sequence tags (ESTs) were obtained by single-pass 5’-end sequencing of the cDNA inserts using cycle-sequencing and dye-terminator standard protocols. The automated capillary electrophoresis sequencing runs were performed on an ABI Prism 3100 (Applied Biosystems). EST sequences were pre-processed using Phred [35]. Only sequences with at least 100 nucleotides and with a Phred quality value greater than or equal to 20 were kept for further analysis. ESTs were screened for vector sequences using the CrossMatch program (http://www.phrap.org). The resulting sequences were assembled into contigs using the CAP3 assembly program [36]. The filtered sequences were compared against the GenBank non-redundant (nr) database using the BLASTX algorithm from the National Center for Biotechnology Information (http://www.ncbi.nlm.nih.gov). Database sequence matches were considered significant at E values ≤ 10^-14^. Transcripts were annotated using Gene Ontology (GO) terms and hierarchical structure (http://www.geneontology.org). Redundancy of the collections of ESTs was calculated as [1-(number of singletons/total number of ESTs)] × 100 [20].

### Quantitative real-time RT-PCR analysis (RT-qPCR)

In this study, RT-qPCR experiments were carried out to check the SSH results and the reliability of our approaches. New independent RNA samples were obtained from the same two conditions: *T. harzianum* grown in FSCW, and *T. harzianum* grown in glucose medium. Both conditions were sampled at three different time points (24, 36 and 48 hours). Statistical tests (Student-*t* test, ANOVA and linear regression analysis) were performed when appropriate. Eighteen genes potentially involved in biocontrol were selected from the FSCW-library and 10 genes from Glc-library (Table 1).

Additional expression analyses were performed by using the dual confrontation plate assay [10]*.* Circular plaques of 5 mm diameter were cut from mycelium of 7-day-old cultures of *T. harzianum* and of *F. solani* grown on MYG plates. *T. harzianum* was inoculated in a distance of 7 cm against *F. solani* mycelium in fresh minimal medium supplemented with 0.2% of glucose plates and overlaid with cellophane. As a control, confrontation assays were conducted following the same procedure, except that *T. harzianum* was challenged against itself. The confrontation plates were incubated in the dark at 28°C and the mycelia were harvested before contact, in the contact, and after contact of the two fungi.

Primers used in RT-qPCR (Table 1) were designed using the PerlPrimer v1.1.20 software. Total RNA was isolated from the mycelia and treated with DNase I (Invitrogen). Total RNA (5 μg) from each sample was reverse transcribed into cDNA in the presence of oligo(dT) primer in a volume of 20 μL using the Revertaid™ First Strand cDNA synthesis kit (Fermentas). The synthesized cDNA was diluted with 80 μL of water and used as a template for RT-qPCR. Reactions were performed in the iQ5 real-time PCR system (Bio-Rad). Each reaction (20 μL) contained 10 μL of MAXIMA® SYBR-green PCR Master mix (Fermentas), forward and reverse primers (500 nM each), cDNA template (5 μg), and nuclease free water. PCR cycling conditions were 10 min at 95°C (1 cycle), 15 s at 95°C followed by 1 min at 60°C (40 cycles), and a melting curve of 1 min at 95°C followed by 30 s at 55°C and a final ramp to 95°C with continuous data collection (1 cycle) to test for primer dimers and nonspecific amplification. The α-tubulin transcripts were used as internal references to normalize gene expression [37].

The expression levels of 28 genes were estimated from the threshold cycle using the 2^-ΔΔCT^ method [38]. In order to evaluate the kinetics of the genes at 24, 36 and 48 hours, the samples were analyzed in three independent experiments with three replicates in each run. Determination of the PCR efficiency was performed using triplicate reactions from a dilution series of cDNA (1, 0.1, 10^-2^ and 10^-3^). Amplification efficiency was then calculated from the given slopes in the IQ5 Optical system Software v2.0 [39]. All calculated efficiencies showed values between 98% and 117%.

## Abbreviations

asm: acid sphingomielinase; aoc: amine oxidase; aqp: aquaporin; cfem: eight cysteine-containing domain; chk1: checkpoint-like protein; chit: chitinase 33; cpox: coproporphyrinogen oxidase; CWDE: Cell Wall Degrading Enzymes; Duf: duf895 domain membrane protein; Endo: Endochitinase 42; Eno: Enolase; Exo: Exo-rhamnogalacturonase; Gapd: Glyceraldehyde 3- phosphate dehydrogenase; Glyc: Glycosyl hydrolase; hsp98: heat shock protein; ht: hexose transporter-like protein; mbl2: mbl2-like secreted; norA: norsolorinic acid reductase; ptr2: peptide transporter; psd: phosphatidylserine decarboxylase family protein; pld: phospholipase d; pdc: pyruvate decarboxylase; qid: QID74 protein; sag: senescence-associated protein; ser: serine protease; sck1: serine threonine-protein kinase; c2h2: zinc finger domain protein; zt: zinc-regulated transporter; bgn: β-1,3-endoglucanase; FSCW-library: *F. solani* cell wall library; Glc-library: Glucose library.

## Competing interests

No competing financial interests exist.

## Authors’ contributions

PMV, ASS and SJLS performed the construction of the cDNA gene libraries and sequenced the ESTs. PMV and ASGC designed the bioinformatics analysis. PMV and RNS performed the RT-PCR analyses and evaluation of the data. PMV drafted the manuscript. ASGC and CJU were responsible for the experimental design and revised the manuscript. All authors approved the final version of the paper.

## Supplementary Material

Additional file 1 **Relative expression profiles of genes identified in *Trichoderma harzianum *(FSCW and Glc libraries) at different times of exposure to *Fusarium solani *cell wall. **The data is presented with log scale for better visualization. Click here for file

Additional file 2 **Relative expression profiles of genes identified *T. harzianum *during interaction with *F. solani. ***The data is presented with log scale for better visualization. Click here for file
