# Supplementary figures and images for: Identification of differentially expressed genes from Trichoderma harzianum during growth on cell wall of Fusarium solani as a tool for biotechnological application
Source: BMC Genomics. 2013 Mar 15;14:177. doi: 10.1186/1471-2164-14-177 (PMC3606605; doi:10.1186/1471-2164-14-177)

■ 24 hours ■ 36 hours ▨ 48 hours

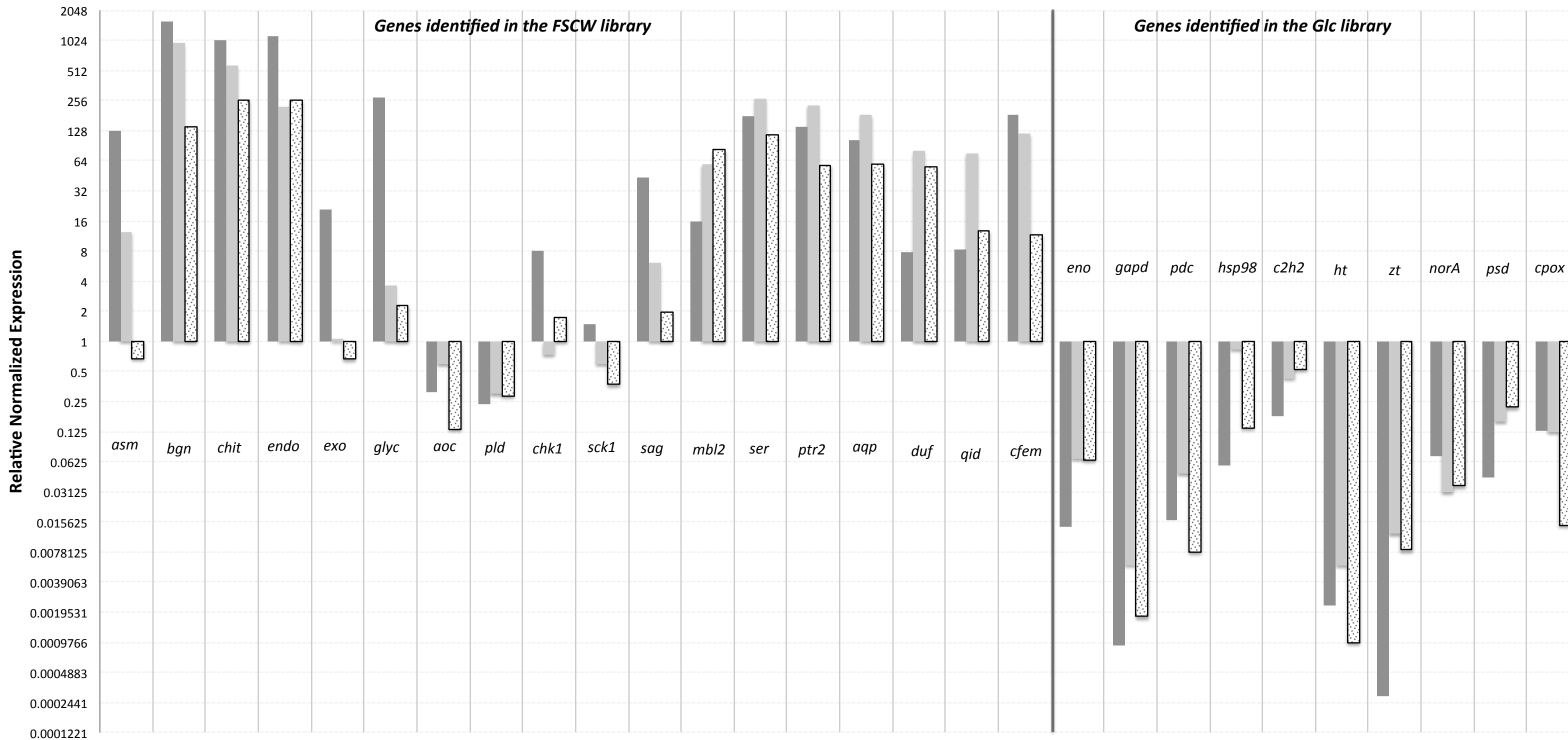

Supplement: Additional file 1 — Relative expression profiles of genes identified in Trichoderma harzianum (FSCW and Glc libraries) at different times of exposure to Fusarium solani cell wall. The data is presented with log scale for better visualization. [file 1471-2164-14-177-S1.pdf]

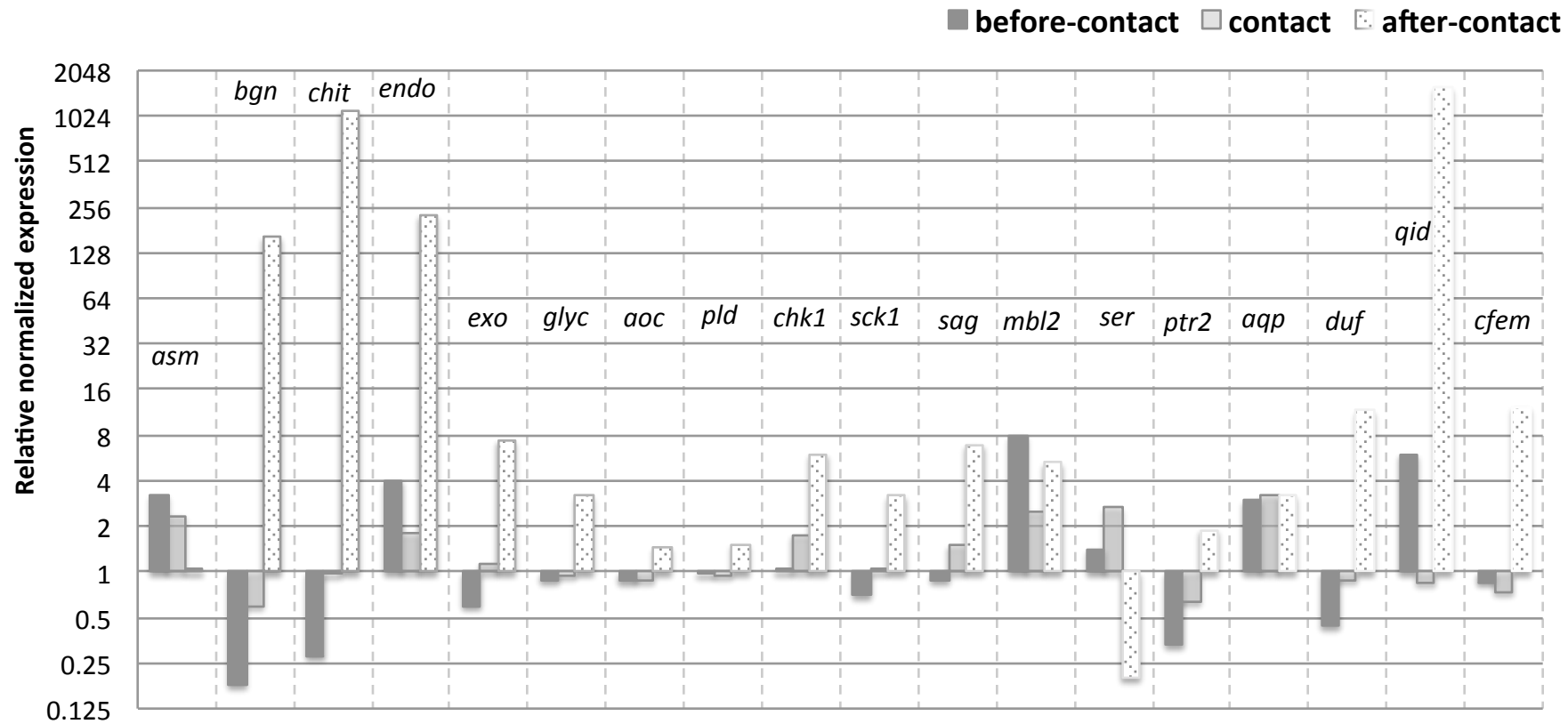

Supplement: Additional file 2 — Relative expression profiles of genes identified T. harzianum during interaction with F. solani. The data is presented with log scale for better visualization. [file 1471-2164-14-177-S2.pdf]
